# Diagnostic performance of transperineal prostate targeted biopsy alone according to the PI-RADS score based on bi-parametric magnetic resonance imaging

**DOI:** 10.3389/fonc.2023.1142022

**Published:** 2023-03-23

**Authors:** Tae Il Noh, Ji Sung Shim, Seok Ho Kang, Jun Cheon, Sung Gu Kang

**Affiliations:** Department of Urology, Anam Hospital, Korea University College of Medicine, Seoul, Republic of Korea

**Keywords:** magnetic resonance imaging, transperineal biopsy, prostate cancer, PI-RADS, target biopsy

## Abstract

**Purpose:**

To compare the diagnostic performance of transperineal targeted biopsy (TB) or systematic biopsy (SB) alone based on combined TB+SB and radical prostatectomy (RP) specimen for detecting prostate cancer (PCa) according to the prostate imaging reporting and data system (PI-RADS) score.

**Materials and methods:**

This study included 1077 men who underwent transperineal bi-parametric (bp) magnetic resonance imaging (MRI)–ultrasound (US) fusion TB+SB (bpMRI-US FTSB) between April 2019 and March 2022. To compare the performance of each modality (TB, SB, and combined TB+SB) with the RP specimen (as the standard) for detecting PCa and clinically significant PCa (csPCa), receiver operating characteristic (ROC) curves were plotted.

**Results:**

PCa was detected in 581 of 1077 men (53.9%) using bpMRI-US FTSB. CsPCa was detected in 383 of 1077 men (35.6%), 17 of 285 (6.0%) with PI-RADS 0 to 2, 35 of 277 (12.6%) with PI-RADS 3, 134 of 274 (48.9%) with PI-RADS 4, and 197 of 241 (81.7%) with PI-RADS 5, respectively. The additional diagnostic value of TB vs. SB compared to combined TB+SB for diagnosing csPCa were 4.3% vs. 3.2% (p=0.844), 20.4% vs 5.1% (p<0.001), and 20.3% vs. 0.7% (p<0.001) with PI-RADS 3, 4, and 5, respectively. TB alone showed no significant difference in diagnostic performance for csPCa with combined TB+SB based on RP specimens in patients with PI-RADS 5 (p=0.732).

**Conclusion:**

A need for addition of SB to TB in patients with PI-RADS 3 and 4 lesions, however, TB alone may be performed without affecting the management of patients with PI-RADS 5.

## Introduction

Prostate cancer (PCa) diagnosis relies on prostate-specific antigen (PSA) and prostate biopsy, and transrectal ultrasonography-guided systematic biopsy (TRUSB) has been considered the standard diagnostic pathway in men with a clinical suspicion of PCa (1).

However, TRUSB has led to missed diagnosis in >30% of patients with PCa and has poor discriminative power in diagnosing cancerous tissue (2, 3). In this regard, to improve the discriminative power and diagnostic accuracy of prostate biopsy, visualization of PCa through magnetic resonance imaging (MRI) has been attempted. Accordingly, the prostate imaging reporting and data system (PI-RADS) was developed to maximize the standardized utilization of MRI for detecting PCa, which led to increased usage of MRI as a guide for targeted biopsy (TB) (4). Studies have suggested that MRI-TB can provide additional value in diagnosis of PCa for clinically significant PCa (csPCa) categorized as International Society for Urological Pathology (ISUP) grade ≥2 (5). Additionally, MRI-TB based on PI-RADS significantly outperforms systematic biopsy (SB) for detection of csPCa with the probability of sparing the potential redundancy of SB (6–8).

However, MRI was missing PCa in 20% of index tumor and 79% of non-index tumor (9). Therefore, the performance of MRI-TB alone may be not good enough to omit systematic biopsy (SB) in every man with a clinical suspicion for PCa (10). TB is the standard pathway in most cancers, nevertheless the current guidelines for detecting PCa have recommended SB and additional TB with a suspicious lesion in MRI (11). However, SB may be associated with over-diagnose the clinically insignificant PCa and result in overtreatment and impose the risk of adverse events, complications, and comes with consequence of medical burden (12, 13). Notably, in PI-RADS 5, MRI-TB have shown good performance with high predictive rates for csPCa that suggests TB alone might also be valuable in diagnosing csPCa (14–16).

The purpose of this study was to compare the diagnostic performance of TB or SB alone according to the PI-RADS scores with combined TB+SB based on the standard transperineal bi-parametric magnetic resolution imaging-ultrasound fusion TB+SB (bpMRI-US FTSB) and radical prostatectomy (RP) specimen.

## Materials and methods

### Study design

**Figure 1:**
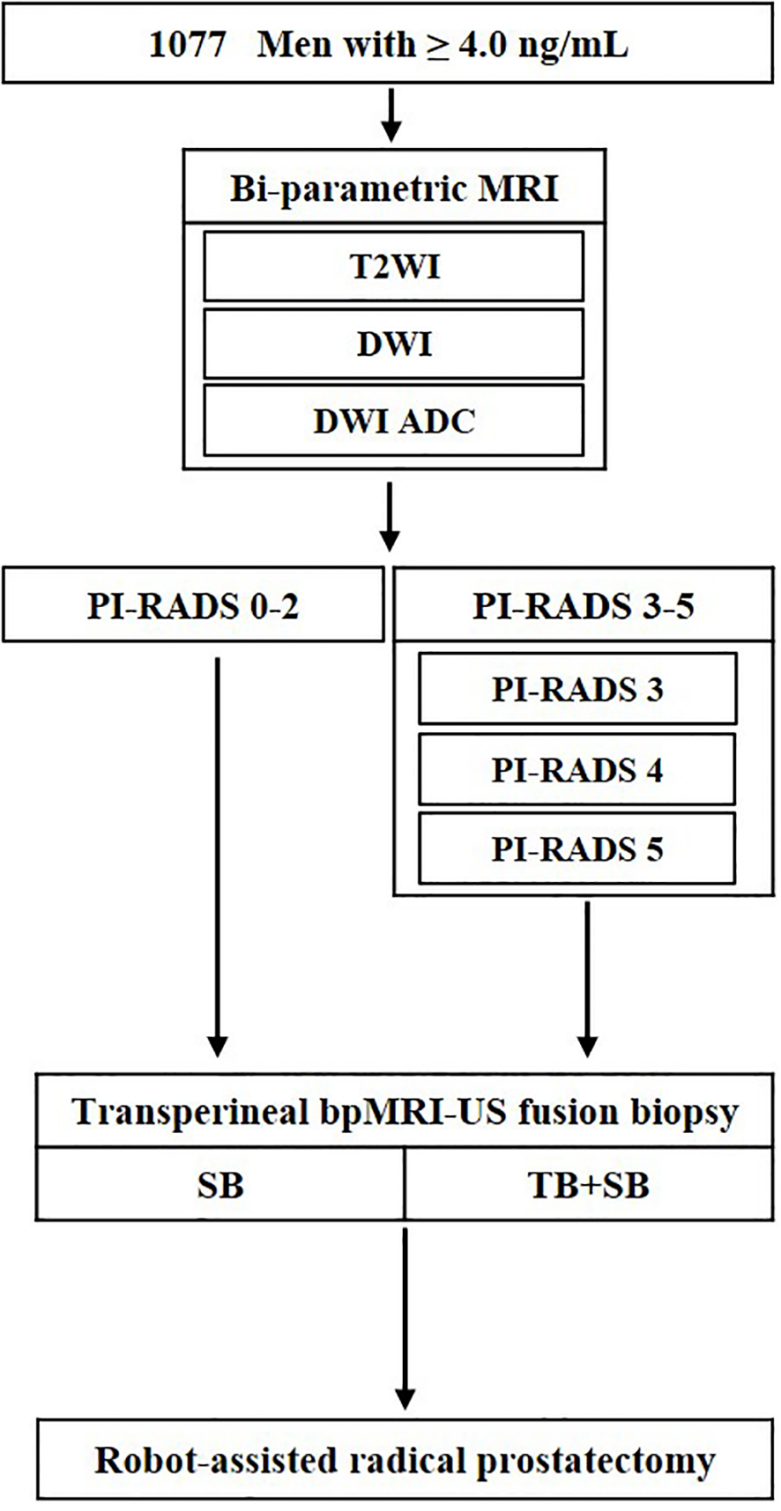
Flowchart of study design. ADC, apparent diffusion coefficient; T2WI, T2-weighted images; DWI, diffusion weighted images; TB, targeted biopsy; SB, systematic biopsy; US, ultrasound.

We analyzed the medical records of 1077 men, between April 2019 and March 2022, who were clinically suspected for PCa with an elevated prostate-specific antigen (PSA) level (≥ 4.0 ng/mL), and/or abnormal findings on digital rectal examination (DRE). All enrolled patients underwent bi-parametric MRI (bpMRI) prior to the prostate biopsy, and regions of interest (ROIs) on MRI were established according to the PI-RADS version 2.0. Subsequent transperineal bpMRI-US FTSB and RP were performed (**Figure 1**).

### MRI acquisition protocol

**Figure 2:**
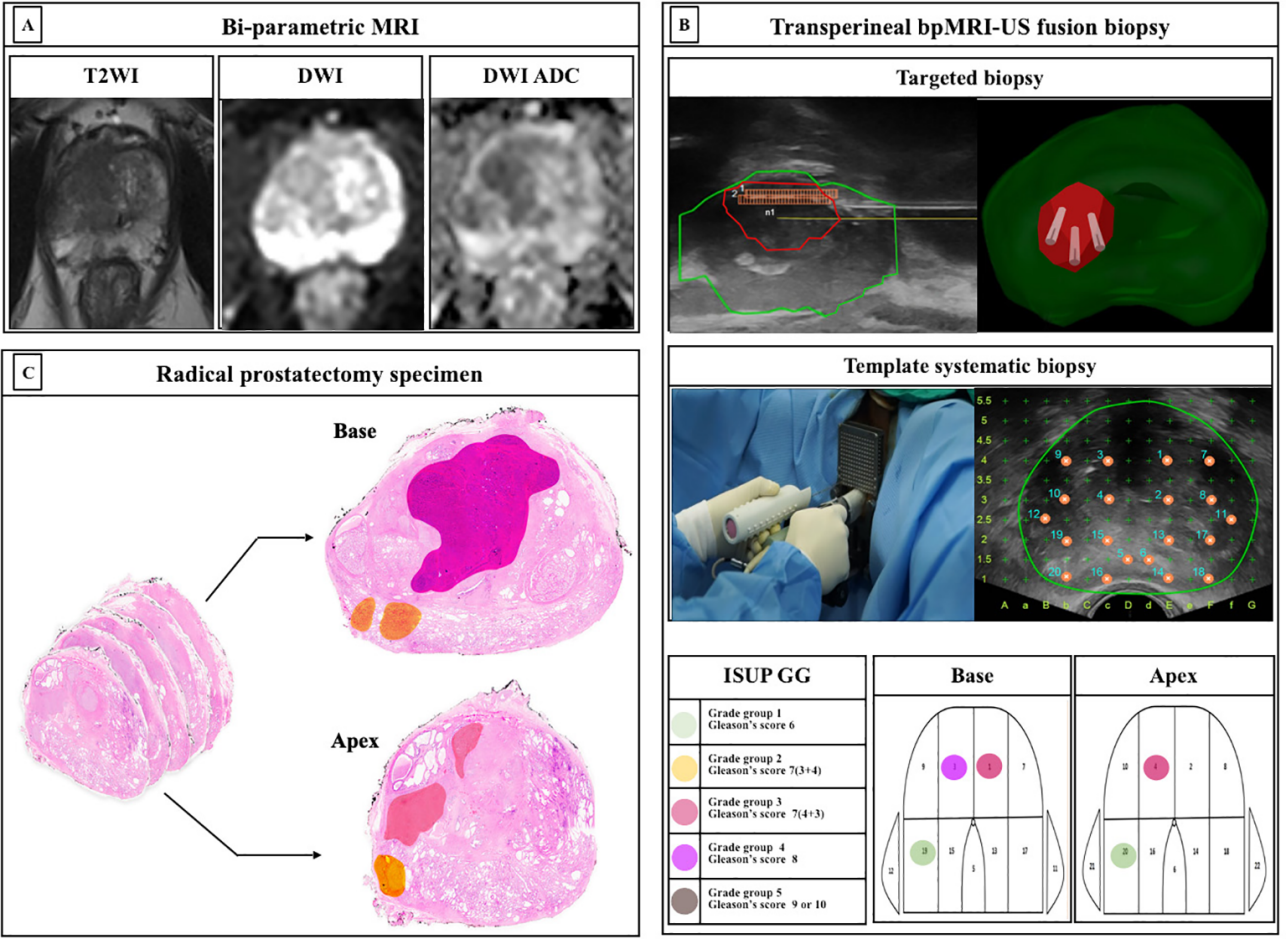
Protocols of study. **(A)** Bi-parametric magnetic resonance imaging (bpMRI) **(B)** Transperineal bpMRI-Ultrasound fusion targeted and systematic biopsy **(C)** Whole-mount radical prostatectomy specimen ISUP, International Society for Urological Pathology; GG, grade group; MRI, Magnetic resonance imaging; TB, targeted biopsy; SB, systematic biopsy.

The bpMRI, contrast-free protocol, was performed using a 3.0-T scanner (Magnetom Skyra and Prisma, Siemens Healthineers, Erlangen, Germany or Achieva, Philips Healthcare, Best, Netherlands) with a multichannel phased-array external surface coil. T2-weighted images (T2WI) and diffusion-weighted images (DWI) were obtained, whereas dynamic contrast-enhanced (DCE) images were omitted. ROIs on the bpMRI were marked by three dedicated uroradiologists based on PI-RADS version 2.0 (**Figure 2A**).

### Prostate biopsy protocol

We have previously reported a protocol for transperineal bpMRI-US FTSB (16). In brief, the elastic image registration type of the MRI-US fusion technique using a mechanical position encoder and robotic articulated arm system (Biojet, USA) was used and TB and SB were performed by urologists during the same session. Further, we considered suspicious lesions as ROI (PI-RADS ≥3) for TB, and 3-4 cores of TB and sequential 22-cores of SB were performed using a prostate mapping template (modified Barzell-template). The ROI for the TB was not intentionally avoided. Each core was labelled separately and subjected to histopathology. The number of biopsy cores was decided depending on the prostate size. The prostate biopsy results were reported by three uropathologists based on the International Society of Urological Pathology (ISUP) grade groups (GG). Clinically insignificant PCa was defined as an ISUP GG1. Clinically significant PCa was defined as > ISUP GG2 (**Figure 2B**).

### RP and histopathologic examination protocol

Localized PCa with PI-RADS 3-5, sequentially underwent robot-assisted RP (RARP) using da Vinci Si, Xi, or SP system (Intuitive Surgical, Sunnyvale, CA, USA) by two surgeons. For histopathological examination, whole-mount histopathology slides were used, and each prostate was sectioned in the axial plane from the basal to the apex at approximately 4-5 mm intervals (**Figure 2C**).

### Study end points

The endpoint was to compare the impact of TB or SB alone according to PI-RADS scores, referring to the standard of combined TB+SB and RP specimens.

### Statistical analysis

To quantify and compare the performance of each modality (TB, SB, and combined TB+SB) in detecting PCa and csPCa, receiver operating characteristic (ROC) curve analyses were performed considering combined TB+SB and RP specimens as standards. Accordingly, the results were summarized using Delong’s test as the areas under the ROC curves (AUCs) and 95% CI. All statistical analyses were performed using IBM SPSS version 26.0 (IBM Corp., Armonk, NY, USA). The level of statistical significance was considered P<0.05.

### Ethics statement

This study was conducted in accordance with the Declaration of Helsinki and current ethical guidelines. The study was reviewed and approved by the Ethics Committee and Institutional Review Board of Korea University Anam Hospital (IRB No. 2018AN0339).

## Result

### Patient demographics

In total, 1077 men were included in the analysis. The median (interquartile range (IQR)) age was 69.0 (62.0-75.0) years. The median (IQR) PSA and PSA density (PSAD) were 6.66 (4.57-11.57) ng/mL and 0.18 (0.11-0.35) ng/mL^2^. The demographics of the study population are reported in **Table 1**.

**Table 1:** Demographics of men according to PI-RADS distribution.

	All	PI-RADS 0-2	PI-RADS 3	PI-RADS 4	PI-RADS 5
Distribution of PI-RADS, n (%)	1077	285 (26.5)	277 (25.7)	274 (25.4)	241 (22.4)
Median Age (IQR)	69.0 (62.0-75.0)	61.0 (56.0-68.0)	66.0 (61.0-72.0)	72.0 (64.8-77.0)	72.0 (68.0-78.0)
Median PSA, ng/mL (IQR)	6.66 (4.57-11.57)	5.27 (4.14-6.73)	5.65 (4.28-8.64)	6.88 (4.89-10.87)	13.3 (7.03-34.3)
Median prostate volume, cm^3^ (IQR)	36.3 (26.4-50.1)	38.9 (27.7-54.0)	39.4 (30.2-51.1)	34.9 (25.3-46.4)	32.1 (24.2-44.4)
Median PSA density (IQR)	0.18 (0.11-0.35)	0.13 (0.08-0.20)	0.15 (0.10-0.22)	0.19 (0.13-0.35)	0.45 (0.22-1.03)
Median free/total PSA ratio (IQR)	0.15 (0.10-0.21)	0.17 (0.12-0.24)	0.17 (0.12-0.23)	0.13 (0.10-0.19)	0.12 (0.08-0.17)
DRE nodule, n (%)	122 (11.3)	15 (5.3)	27 (9.7)	42 (15.3)	38 (15.8)

PI-RADS, prostate imaging-reporting and data systems; IQR, interquartile range; PSA, prostate-specific antigen; DRE, digital rectal exam.

### Diagnostic performance of bpMRI-US FTSB

**Figure 3:**
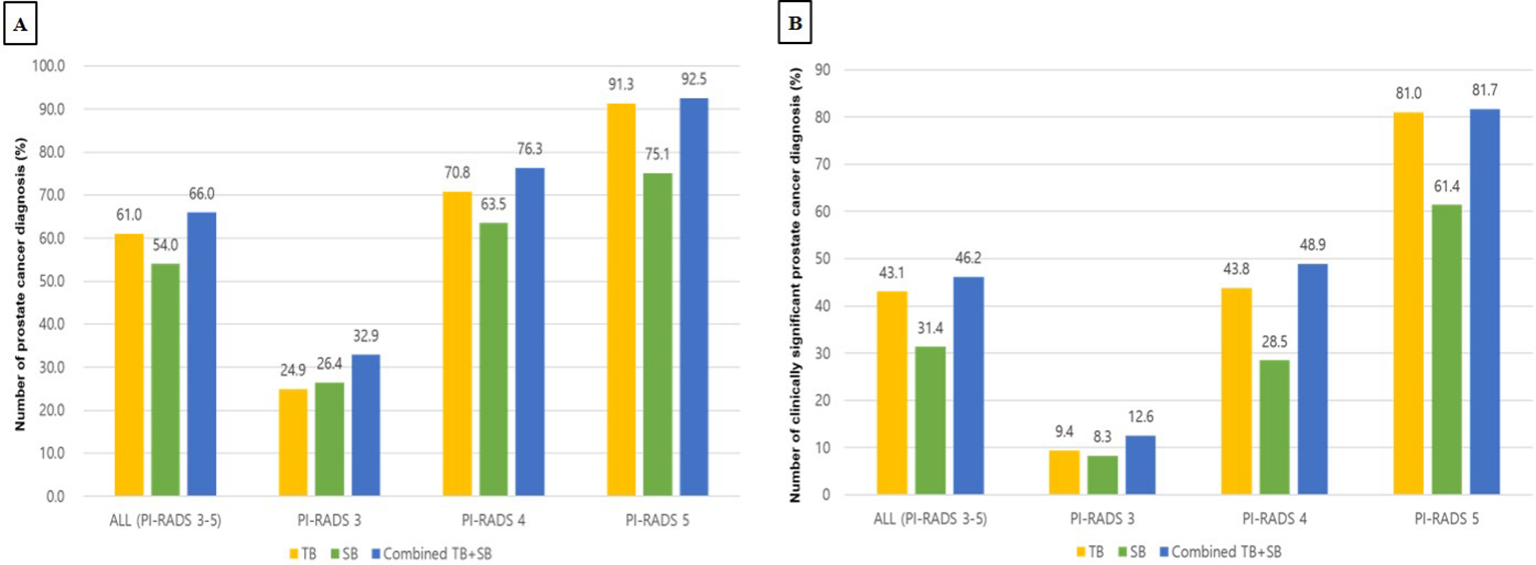
Diagnostic performance of TB, SB, TB+SB in patients with PI-RADS 3 to 5 **(A)** Detection rate for prostate cancer **(B)** Detection rate for clinically significant prostate cancer PI-RADS, prostate imaging-reporting and data systems; TB, targeted biopsy; SB, systematic biopsy.

PCa (GG1) was detected in 581 of 1077 men (53.9%) by bpMRI-US FTSB. Accordingly, it was detected in 58 of 285 cases (35.6%) with PI-RADS 0-2, in 91 of 277 cases (32.9%) with PI-RADS 3, in 209 of 274 cases (76.3%) with PI-RADS 4, and in 220 of 241 cases (91.3%) with PI-RADS 5 (**Figure 3A**). Further, csPCa (≥ GG2) was detected in 383 of 1077 men (35.6%). Accordingly, it was detected in 17 of 285 men (6.0%) with PI-RADS 0-2, in 35 of 277 men (12.6%) with PI-RADS 3, in 134 of 274 men (48.9%) with PI-RADS 4, and in 197 of 241 men (81.7%) with PI-RADS 5 (**Figure 3B**). The distribution of ISUP grade groups is shown in **Table 2**. Patients with csPCa (GG2≥2) had higher median PSA, PSAD, and lower prostate volume than those with GG1 pathology; PSA(IQR) [66.0 vs. 72.0, p= 0.038], PSAD (0.14 vs. 0.35, p=0.011), and lower prostate volume (41.2 vs. 30.3, p=0.047) than those with GG1 pathology (**Supplementary Table 1**).

**Table 2:** Diagnostic performance of transperineal MRI-US fusion TB and SB.

	All	PI-RADS 0-2	PI-RADS 3			PI-RADS 4			PI-RADS 5		
	1077	285	277			274			241		
		SB	TB	SB	TB+SB	TB	SB	TB+SB	TB	SB	TB+SB
**PCa, n (%)**	581 (53.9)	58 (20.4)	69 (24.9)	73 (26.4)	91 (32.9)	194 (70.8)	174 (63.5)	209 (76.3)	220 (91.3)	181 (75.1)	223 (92.5)
**csPCa, n (%)**	383 (35.6)	17 (6.0)	26 (9.4)	23 (8.3)	35 (12.6)	120 (43.8)	78 (28.5)	134 (48.9)	195 (81.0)	148 (61.4)	197 (81.7)
ISUP*, n (%)											
1	198 (34.1)	41 (14.4)	43 (15.5)	50 (18.1)	56 (20.2)	74 (27.0)	96 (35.0)	75 (27.4)	25 (10.4)	33 (13.7)	26 (10.8)
2	119 (20.5)	10 (3.5)	15 (5.4)	17 (6.1)	22 (7.9)	43 (15.7)	25 (9.1)	44 (16.1)	46 (19.1)	42 (17.4)	43 (17.8)
3	37 (6.4)	5 (1.8)	3 (1.1)	1 (0.4)	2 (0.7)	11 (4.0)	7 (2.6)	15 (5.5)	28 (11.6)	12 (5.0)	15 (6.2)
4	180 (30.9)	1 (0.4)	8 (2.9)	5 (1.8)	11 (4.0)	60 (21.9)	41 (15.0)	66 (24.1)	91 (37.8)	66 (27.4)	102 (42.3)
5	47 (8.1)	1 (0.4)	0 (0.0)	0 (0.0)	0 (0.0)	6 (2.2)	5 (1.8)	9 (3.3)	30 (12.4)	28 (11.6)	37 (15.4)

* ISUP grade groups (GG):1 = Gleason 6 (or less), 2 = Gleason 7(3 + 4), 3 = Gleason 7(4 + 3), 4 = Gleason 8(4 + 4 or 3 + 5 or 5 + 3), and 5 = Gleason 9 or 10. csPCa: ≥ ISUP GG2.

MRI-US, magnetic resonance imaging-ultrasonography; ISUP, International Society for Urological Pathology; TB, targeted biopsy; SB, systematic biopsy; PCa, prostate cancer; PI-RADS, prostate imaging reporting and data system.

### Comparison of the diagnostic performance of TB or SB alone with the standard of combined TB+SB

In patients with PI-RADS 3 to 5, TB, SB, and Combined TB+SB were able to detect PCa in 61.0%, 54.0%, and 66.0% of cases, respectively. Accordingly, the diagnosis rate of TB, SB, and combined TB+SB for diagnosing PCa were 24.9%, 26.4%, and 32.9% in patients with PI-RADS 3, 70.8%, 63.5%, and 76.3% in patients with PI-RADS 4, and 91.3%, 75.1%, and 92.5% in patients with PI-RADS 5, respectively (**Figure 3A**). The additional diagnostic value for PCa detection of TB vs. SB compared to combined TB+SB were 12.0% vs. 5.0% (p<0.001) in patients with PI-RADS 3-5; PI-RADS 3: 6.5% vs. 8.0% (p=0.535), PI-RADS 4: 12.8% vs. 5.5% (p<0.001), and PI-RADS 5: 17.4% vs. 1.2% (p<0.0001), respectively (**Table 3**).

**Table 3:** Diagnostic performance of TB or SB alone according to PI-RADS compared to combined TB and SB.

	All (PI-RADS 3-5)			PI-RADS 3			PI-RADS 4			PI-RADS 5		
	792			277			274			241		
	TB	SB	TB+SB	TB	SB	TB+SB	TB	SB	TB+SB	TB	SB	TB+SB
PCa, n (%)	483 (61.0)	428 (54.0)	523 (66.0)	69 (24.9)	73 (26.4)	91 (32.9)	194 (70.8)	174 (63.5)	209 (76.3)	220 (91.3)	181 (75.1)	223 (92.5)
Additional value of TB	12.0%			6.5 %			12.8 %			17.4 %		
Additional value of SB	5.0%			8.0 %			5.5 %			1.2 %		
AUC (CI 95%) Reference to TB+SB	0.932 (0.915-0.947)	0.914 (0.895-0.931)		0.882 (0.838-0.918)	0.904 (0.863-0.937)		0.964 (0.935-0.983)	0.916 (0.877-0.946)		0.986 (0.961-0.997)	0.906 (0.862-0.940)	
p value (vs. TB+SB)	<0.001	<0.001		<0.001	<0.001		<0.001	<0.001		0.078	<0.001	
csPCa (≥ GG2) *, n (%)	341 (43.1)	249 (31.4)	366 (46.2)	26 (9.4)	23 (8.3)	35 (12.6)	120 (43.8)	78 (28.5)	134 (48.9)	195 (81.0)	148 (61.4)	197 (81.7)
Additional value of TB	14.8%			4.3%			20.4%			20.3%		
Additional value of SB	3.1%			3.2%			5.1%			0.7%		
AUC (CI 95%) Reference to TB+SB	0.957 (0.942-0.968)	0.881 (0.895-0.901)		0.893 (0.851-0.927)	0.883 (0.839-0.918)		0.961 (0.931-0.981)	0.841 (0.792-0.882)		0.989 (0.971-0.998)	0.867 (0.817-0.907)	
p value (vs. TB+SB)	<0.001	<0.001		0.009	0.004		0.0021	<0.001		0.093	<0.001	

* ISUP grade groups (GG):1 = Gleason 6 (or less), 2 = Gleason 7(3+4), 3 = Gleason 7(4+3), 4 = Gleason 8(4+4 or 3+5 or 5+3), and 5 = Gleason 9 or 10. csPCa: ≥ ISUP GG2

AUC, area under the curve; CI, confidence interval; ISUP, International Society for Urological Pathology; TB, targeted biopsy; SB, systematic biopsy; PCa, prostate cancer; csPCa, clinically significant prostate cancer; PI-RADS, prostate imaging reporting and data system.

Combined TB+SB showed superior diagnostic performance for TB or SB alone in patients with PI-RADS 3 and 4 (p <0.001). However, TB alone showed no significant difference in diagnostic performance for csPCa with combined TB+SB in patients with PI-RADS 5; PI-RADS 3: area under the curve (AUC) [95% confidence interval (CI)], 0.882 [0.838–0.918], p<0.001; PI-RADS 4: AUC, 0.964 [0.935–0.983], p<0.001; PI-RADS 5: AUC, 0.986 [0.961–0.997], p=0.078 (**Table 3**).

In patients with PI-RADS 3 to 5, csPCa (ISUP ≥GG2) was detected in 43.1%, 31.4%, and 46.2% cases *via* TB, SB, and combined TB+SB, respectively. Accordingly, the diagnosis rate of TB, SB, and combined TB+SB for diagnosing csPCa were 9.4%, 8.3%, and 12.6% in patients with PI-RADS 3, 43.8%, 28.5%, and 48.9% in patients with PI-RADS 4, and 81.0%, 61.4%, and 81.7% in patients with PI-RADS 5, respectively (**Figure 3B**). The additional diagnostic value for csPCa detection of TB vs. SB alone compared to combined TB+SB was 14.8% vs. 3.1%(p<0.001) in patients with PI-RADS 3-5; PI-RADS 3: 4.3% vs. 3.2% (p=0.844), PI-RADS 4: 20.4% vs. 5.1% (p<0.001), and PI-RADS 5: 20.3% vs. 0.7% (p<0.001), respectively (**Table 3**). Further, TB alone showed no significant difference in diagnostic performance for csPCa to combined TB+SB in patients with PI-RADS 5; PI-RADS 3: area under the curve (AUC) [95% confidence interval (CI)], 0.893 [0.851–0.927], p=0.0088; PI-RADS 4: AUC, 0.961 [0.931–0.981], p=0.002; PI-RADS 5: AUC, 0.989 [0.971–0.998], p=0.093 (**Table 3**).

### Comparison of diagnostic performances referring to RP specimen

The RARP was performed in 289 of 483 diagnosed with PCa with PI-RADS 3-5; 59 of 91 (64.8%) with PI-RADS 3, 122 of 209 (58.4%) with PI-RADS 4, and 108 of 220 (49.1%) with PI-RADS 5, respectively (**Table 4**).

**Table 4:** Concordance of prostate cancer grade group on targeted, systematic, and combined targeted and systematic biopsy according to radical prostatectomy specimen by PI-RADS scores.

	Radical prostatectomy, n (%)											
	All (PI-RADS 3-5)			PI-RADS 3			PI-RADS 4			PI-RADS 5		
	289			59			122			108		
	TB	SB	TB+SB	TB	SB	TB+SB	TB	SB	TB+SB	TB	SB	TB+SB
**PCa**	265 (91.7)	239 (82.7)	289 (100.0)	42 (71.2)	47 (79.7)	59 (100.0)	115 (94.2)	104 (85.2)	122 (100.0)	108 (100.0)	84 (77.8)	108 (100.0)
Any upgrading of GG*	121 (45.7)	168 (70.3)	96 (33.2)	32 (76.2)	35 (74.5)	22 (37.3)	58 (50.4)	79 (76.0)	48 (39.3)	31 (28.7)	54 (64.3)	26 (24.1)
Any downgrading of GG*	73 (27.5)	55 (23.0)	93 (32.2)	6 (14.3)	3 (6.4)	9 (15.3)	30 (26.1)	24 (23.1)	37 (30.3)	37 (34.3)	28 (33.3)	47 (43.5)
GG1*	79 (29.8)	113 (47.3)	89 (30.8)	26 (61.9)	33 (70.2)	36 (61.0)	44 (38.3)	62 (59.6)	45 (36.9)	9 (8.3)	23 (27.9)	8 (7.4)
Upgrading GG1 to GG ≥ 2	59 (22.3)	94 (39.3)	59 (20.4)	17 (40.5)	21 (44.7)	21 (35.6)	38 (33.0)	54 (51.9)	35 (28.7)	4 (3.7)	19 (22.6)	3 (2.8)
GG ≥ 2*	186 (70.2)	126 (52.7)	200 (69.2)	16 (38.1)	14 (29.8)	23 (39.0)	71 (61.7)	42 (40.4)	77 (63.1)	99 (91.7)	66 (78.6)	100 (92.6)
Downgrading GG ≥ 2 to GG 1	1 (0.4)	0 (0.0)	1 (0.3)	1 (2.3)	0 (0.0)	1 (1.7)	0 (0.0)	0 (0.0)	0 (0.0)	0 (0.0)	0 (0.0)	0 (0.0)
AUC (CI 95%) Reference to RP specimen	0.824 (0.777-0.864)	0.719 (0.665-0.768)	0.860 (0.809-0.911)	0.663 (0.524-0.802)	0.667 (0.531-0.802)	0.722 (0.593-0.852)	0.817 (0.730-0.904)	0.688 (0.559-0.816)	0.844 (0.766-0.921)	0.951 (0.909-0.994)	0.820 (0.711-0.929)	0.961 (0.924-0.998)
p value (vs. TB+SB)	0.034	<0.001		0.016	0.021		0.049	<0.001		0.732	<0.001	

* ISUP grade groups (GG):1 = Gleason 6 (or less), 2 = Gleason 7(3 + 4), 3 = Gleason 7(4 + 3), 4 = Gleason 8(4 + 4 or 3 + 5 or 5 + 3), and 5 = Gleason 9 or 10. csPCa: ≥ ISUP GG2.

AUC, area under the curve; CI, confidence interval; ISUP, International Society for Urological Pathology; TB, targeted biopsy; SB, systematic biopsy; PCa, prostate cancer; csPCa, clinically significant prostate cancer; PI-RADS, Prostate Imaging Reporting and Data System.

Accordingly, TB alone and combined TB+SB showed 45.7% and 33.2% of any upgrading in RP specimens with PI-RADS 3-5; 76.2% and 37.3% with PI-RADS 3, 50.4% and 39.3% with PI-RADS 4, 28.7% and 24.1% with PI-RADS 5, respectively; and upgrading of GG1 to GG ≥ 2 occurred in 59 of 265 (22.3%) and 59 of 289 (20.4%) cases with PI-RADS 3-5; 17 of 42 (40.5%) and 21of 59 (35.6%) with PI-RADS 3, 38 of 115 (33.0%) and 35 of 122 (28.7%) with PI-RADS 4, and 4 of 108 (3.7%) and 3 of 108 (2.8%) with PI-RADS 5, respectively. Further, downgrading of GG ≥ 2 to GG1 occurred in only one in 289 (0.3%) (**Table 4**).

The combined TB+SB showed superior diagnostic performance compared to TB alone for diagnosing csPCa when compared to the standard of RP specimen; TB alone vs TB+SB, AUC (95% CI); PI-RADS 3-5: 0.824 (0.777-0.864) vs. 0.860 (0.809-0.911), p=0.034; PI-RADS 3: 0.663 (0.524-0.802) vs. 0.722 (0.593-0.852), p=0.016; PI-RADS 4: 0.817 (0.730-0.904) vs. 0.844 (0.766-0.921), p=0.049. TB alone showed no significant difference in diagnostic performance for csPCa to combined TB+SB in patients with PI-RADS 5; TB alone vs. combined TB+SB, AUC (95% CI), 0.951(0.909-0.994) vs. 0.961(0.924-0.998), p=0.732 (**Table 4**).

## Discussion

In recent years with significant improvements in the accuracy of MRI after implementation of the PI-RADS, the use of prebiopsy MRI for PCa diagnosis has increased (4, 6, 17). Furthermore, numerous studies have demonstrated that MRI-TB could offer improved diagnostic value for csPCa with pooled sensitivity and specificity of 0.80 (95%CI: 0.69-0.87) and 0.94 (95%CI: 0.90-0.97) (5). However, addition of TB to SB increases the number of csPCa (≥ ISUP GG2) by 6.7-7.6%, while added value of SB to TB is 4.3-5.2% for csPCa (5, 14, 18). Further, MRI was missing PCa in 20% of index tumor and 79% of non-index tumor (9). Therefore, due to the additional diagnostic value of SB and the risk of missing csPCa with TB alone, combined TB + SB has been suggested for dignosing PCa (5, 10, 11).

However, it should be noted that obtaining more prostate cores accompanies with a greater risk of complications, such as prostatitis, sepsis events, visits to the emergency room, rectal bleeding, hematuria, and pain (7, 19, 20). MRI-TB alone with fewer core biopsies per patient might lead to fewer complications. The net benefit of adding SB to TB for prostate biopsy optimization according to PI-RADS score should be weighed against accuracy for csPCa detection and additional burden such as overdiagnosis of indolent PCa, resulting in overtreatment and complications from increased numbers of biopsies. For predicting csPCa, several predictors and their combination such as clinical parameters including PSAD and PI-RADS score have been suggested (21). In addition, for risk assessment to determine the need for biopsy, risk calculators (RCs) have been suggested, thereby may be reducing the number of unnecessary biopsies (22).

Notably, MRI-TB showed good performance and was highly predictive for diagnosing csPCa in cases with PI-RADS 5 (77-85%) (7, 14, 16). In a study comparing the concordances between PI-RADS and histologic reports of the RP specimen, the PI-RADS≥3 was further associated with csPCa in 92.4% of cases, with 100% association in cases with a PI-RADS 5 score (23). High performance of MRI-TB and low additional diagnostic value (2-4%) of SB for detection of csPCa in patients with PI-RADS 5 that suggests the probability of sparing the potential redundancy of SB in PI-RADS 5 (12, 24, 25).

For MRI-TB, mpMRI have shown a high sensitivity and negative predictive value (NPV) of 93.0% and 89.0% for csPCa (6). However, it is time-consuming (~ 40 min) to acquire T2-weighted imaging (T2WI) and diffusion-weighted imaging (DWI), and dynamic contrast-enhanced (DCE) imaging requires intravenous administration of contrast media.

Several studies have demonstrated comparable diagnostic performance of bpMRI (contrast-free protocol) to mpMRI (26). In a systematic review and meta-analysis of the diagnostic accuracy of bpMRI and mpMRI for PCa detection, pooled sensitivity and specificity did not show significant difference and the AUCs were similar; 0.87 and 0.90 for mpMRI and bpMRI (27). In this regard, bpMRI is more rapid (~15 min) due to exclusion of DCE, and safer from potential side effects of contrast media than mpMRI while retaining a sufficient diagnostic value (16).

In the current study, we compared the impact of TB, SB, and combined TB+SB according to the PI-RADS score. Accordingly, the SB had only additional diagnostic values of 1.2% and 0.7% for detection of PCa and csPCa in patients with PI-RADS 5. Further, TB alone showed no significant difference of diagnostic performance with combined TB+SB for csPCa. Similarly, in a study conducted on 112 patients with PI-RADS 5 on MRI and subsequentially 78 of RP, TB alone could diagnose PCa with very high probability (97%) in patients with PSAD >0.15ng/ml^2^ (12). Accordingly, if SB was omitted, none of the PCa cases and only 4% of csPCa cases would be missed. Thus, the authors suggest that SB might be omitted for cases with PI-RADS 5 and PSAD >0.15ng/ml^2^.

Since the upgrading grade group of RP specimens from prostate biopsy has been reported, the omission of SB may lead to misclassification of PCa; TB (30.9%) and TB+SB (14.4%) of the upgraded grade group (10). These inconsistencies between biopsy and specimen of prostate, upgrading and misclassification of PCa, are the inherent limitations of prostate needle biopsy (28). Nevertheless, in this study, upgrading from GG1 to ≥ GG2, which has a potential risk of changing subsequent clinical management, showed difference in only one patient; TB alone vs. combined TB+SB, 4 of 108 (3.7%) vs. 3 of 108 (2.8%). Similarly, in another study, MRI-TB alone in PI-RADS 5 cases had meager upgrade rate (3.4%) (29). Further, addition of SB to TB in PI-RADS 5 cases altered only 3.1% of the highest grade group of PCa patients, all of whom had already been categorized as GG≥2 based on TB, and SB did not change subsequent clinical management (24). Current study supports the need for SB in patients with PI-RADS 3 and 4 lesions. However, minimal additional diagnostic values of SB and comparable diagnostic performance of MRI-TB suggest that SB potentially can be omitted in patients with PI-RADS 5.

The limitations of this study are its retrospective nature and accompanying bias. The other limitation is that this study was performed in a single tertiary center with transperineal prostate biopsy and bpMRI, and transrectal prostate biopsy with mpMRI, which is the common practice, was not considered. This may raise concerns toward extrapolating a general trend from our results. Nevertheless, this study can support that performing TB alone in patients with PI-RADS 5 lesions, might mitigate the medical burden by SB omission.

## Conclusion

The current study suggests a need for addition of SB to TB in patients with PI-RADS 3 and 4 lesions, and TB alone may be performed for diagnosing csPCa in patients with PI-RADS 5, without changing the subsequent clinical management.

## Data availability statement

The original contributions presented in the study are included in the article/**Supplementary Material**. Further inquiries can be directed to the corresponding author.

## Ethics statement

This study was approved by the Ethics Committee and the Institutional Review Board of KUMC (IRB No. 2018AN0339).

## Author contributions

TIN: protocol/project development, data acquisition, data analysis and interpretation, drafting of the manuscript. JSS: Protocol/project development, supervision. SHK: Protocol/project development, Data acquisition, supervision. JC: Protocol/project development, supervision. SGK: Protocol/project development, Data acquisition, supervision. All authors contributed to the article and approved the submitted version.
