# Supplementary material for: Diagnostic performance of transperineal prostate targeted biopsy alone according to the PI-RADS score based on bi-parametric magnetic resonance imaging
Source: Front Oncol. 2023 Mar 23;13:1142022. doi: 10.3389/fonc.2023.1142022 (PMC10080665; doi:10.3389/fonc.2023.1142022)
Supplement: Supplementary file 1 [file Table_1.docx]

Supplementary Table 1. Demographics of men according to PI-RADS distribution and ISUP grade groups

|  | **All (PI-RADS 0-5)** | | | **PI-RADS 0-2** | | | **PI-RADS 3** | | | **PI-RADS 4** | | | **PI-RADS 5** | | |
| --- | --- | --- | --- | --- | --- | --- | --- | --- | --- | --- | --- | --- | --- | --- | --- |
| **Distribution, n(%)** | 1077 (100.0) | | | 285 (26.5) | | | 277 (25.7) | | | 274 (25.4) | | | 241 (22.4) | | |
|  | GG1 | ≥ GG2 | p value | GG1 | ≥ GG2 | p value | GG1 | ≥ GG2 | p value | GG1 | ≥ GG2 | p  value | GG1 | ≥ GG2 | p value |
| **ISUP grade group**, n (%)** | 198  (34.1) | 383  (65.9) |  | 41 (14.4) | 17 (6.0) |  | 56 (20.2) | 35 (12.6) |  | 75 (27.4) | 134 (48.9) |  | 26 (10.8) | 197 (81.7) |  |
| Median Age (IQR) | 66.0  (60.0-72.0) | 72.0  (67.0-78.0) | 0.038 | 61.0  (56.0-67.0) | 68.5  (58.0-73.5) | 0.023 | 66.0  (60.5-72.0) | 70.0  (65.0-73.0) | 0.294 | 70.5  (63.0-75.0) | 73.0  (65.0-78.0) | 0.690 | 71.0  (63.7-71.0) | 73.0  (68.0-79.0) | 0.318 |
| Median PSA, ng/mL (IQR) | 5.46  (4.12-8.05) | 10.7  (6.16-22.4) | 0.006 | 4.94  (4.12-6.81) | 5.64  (4.62-6.21) | 0.046 | 5.35  (4.15-8.27) | 7.60  (4.63-10.9) | 0.012 | 6.19  (4.63-9.35) | 7.96  (5.19-13.2) | 0.036 | 6.04  (4.22-9.77) | 17.9  (8.82-41.5) | <0.001 |
| Median prostate volume, cm^3^ (IQR) | 41.2  (30.5-55.6) | 30.3  (23.6-40.6) | 0.047 | 39.4  (28.6-54.7) | 37.6  (23.5-39.6) | 0.170 | 41.2  (32.1-55.2) | 29.6  (22.6-39.2) | 0.026 | 41.2  (28.9-54.1) | 29.5  (23.6-37.8) | 0.029 | 34.5  (25.9-51.4) | 31.1  (23.8-42.9) | 0.584 |
| Median PSA density (IQR) | 0.14  (0.09-0.20) | 0.35  (0.20-0.74) | 0.011 | 0.12  (0.08-0.19) | 0.16  (0.13-0.22) | 0.041 | 0.14  (0.09-0.20) | 0.24  (0.16-0.33) | 0.016 | 0.15  (0.11-0.24) | 0.27  (0.17-0.44) | <0.001 | 0.17  (0.10-0.27) | 0.52  (0.28-1.16) | <0.001 |
| Median free/total PSA ratio (IQR) | 0.17  (012-0.23) | 0.11  (0.08-0.16) | 0.076 | 0.17  (012-0.24) | 0.13  (0.11-0.17) | 0.176 | 0.18  (0.12-0.24) | 0.13  (0.10-0.18) | 0.087 | 0.16  (0.11-0.22) | 0.11  (0.09-0.14) | 0.084 | 0.16  (0.11-0.22) | 0.12  (0.08-0.17) | 0.131 |

* ISUP grade groups (GG):1 = Gleason 6 (or less), 2 = Gleason 7(3+4), 3 = Gleason 7(4+3), 4 = Gleason 8(4+4 or 3+5 or 5+3), and 5 = Gleason 9 or 10. csPCa: ≥ ISUP GG2
