# Supplementary material for: Diagnostic performance of transperineal prostate targeted biopsy alone according to the PI-RADS score based on bi-parametric magnetic resonance imaging
Source: Front Oncol. 2023 Mar 23;13:1142022. doi: 10.3389/fonc.2023.1142022 (PMC10080665; doi:10.3389/fonc.2023.1142022)
Supplement: Supplementary file 2 [file Table_2.docx]

| Supplementary Table 2. **The complication of bpMRI-US transperineal FTSB** | | | |
| --- | --- | --- | --- |
| **Complication*** |  | **N (%)** |  |
| **Negative** |  | 761 (70.7) |  |
| **Positive**** |  | 316 (29.3) |  |
| Grade 1 | Self-regression Hematuria | 239 (22.1) |  |
| Grade 2 | Acute urinary retention | 77 (7.1) |  |

* Clavien–Dindo classification; Grade 1-2: minor complications, Grade 3-5: severe complications; Grade 5: Motality

** There were no complications over Grade 3
